# Influencing Mechanisms and Additive-Mediated Modulation of Aggregation Behavior in Papain-Catalyzed Soy Protein Hydrolysis

**DOI:** 10.3390/molecules31213864

**Published:** 2026-10-26

**Authors:** Hongyang Pan, Zhaojun Wang, Xiaofang Chu, Jie Chen

**Affiliations:** 1 State Key Laboratory of Food Science and Resources, Jiangnan University, Wuxi 214122, China; rickypan@jiangnan.edu.cn (H.P.); zhaojun.wang@jiangnan.edu.cn (Z.W.); 2 Analysis and Testing Center, Jiangnan University, Wuxi 214122, China; 3 Institute of Botany, Jiangsu Province and Chinese Academy of Sciences, Nanjing 210014, China; cxf1002@163.com

**Keywords:** soy protein isolate, protease, aggregation mechanism, sulfhydryl groups, protein hydrolysis

## Abstract

Protein aggregation during enzymatic hydrolysis detrimentally impacts product quality. However, due to the inherent complexity of soy protein systems, their precise aggregation mechanisms remain elusive. In this study, papain was employed to hydrolyze soy protein isolate (SPI), as well as 7S and 11S globulins, to elucidate the underlying aggregation mechanisms and explore potential regulatory strategies. The solubility characteristics of the resulting aggregates were evaluated via turbidity measurements (OD660) following treatments with varying concentrations of urea, guanidine hydrochloride, sodium dodecyl sulfate (SDS), and β-mercaptoethanol (β-Me). Amino acid composition and sulfhydryl determination revealed that the turbidity of aggregates decreased with increasing concentrations of urea, guanidine hydrochloride, and SDS, indicating that hydrophobic interactions are a major contributing factor to aggregation. Hydrophobic amino acids such as phenylalanine and leucine were enriched in aggregates compared with the supernatant, further supporting the association of hydrophobic interactions. Sulfhydryl analysis showed that total sulfhydryl content increased in hydrolyzed 11S but decreased in 7S, with higher sulfhydryl levels detected in precipitates than in supernatants, suggesting that disulfide bonds exhibit an auxiliary contribution to 11S aggregation. To regulate aggregation, Na_2_SO_3_ (0.1%), cysteine, or SDS was added prior to enzyme inactivation. Among these, SDS produced the most significant improvement in supernatant yield (18.77%, 12.57%, and 10.73% for SPI, 7S, and 11S, respectively), primarily by disrupting hydrophobic interactions. Cysteine, as a strong reducing agent, was more effective in suppressing 11S aggregation through the targeted cleavage of disulfide bonds. Tricine-SDS-PAGE confirmed that aggregates were mainly composed of small peptides linked through hydrophobic interactions, with disulfide bonds providing partial stabilization, while both SDS and cysteine effectively inhibited aggregate formation. Overall, hydrophobic interactions were identified as a major correlative factor driving soy protein aggregation during enzymatic hydrolysis, with disulfide bonds playing an auxiliary role in 11S aggregation. These findings provide a robust theoretical basis for the regulation of protein hydrolysate aggregation.

## 1. Introduction

Protein or peptide aggregation is a common phenomenon during food processing and can negatively influence product quality by reducing solubility, altering texture, and decreasing nutritional bioavailability [1]. Native soy protein systems predominantly comprise two major storage globulins, β-conglycinin (7S) and glycinin (11S), which account for approximately 70% of the total protein content. 7S is a trimeric glycoprotein, while 11S is a hexameric protein stabilized by disulfide bonds [2]. Consequently, it is crucial to characterize the structural features of protein aggregates, determine the intermolecular forces responsible for their formation, and elucidate the mechanisms underlying aggregation induced by enzymatic hydrolysis. Due to the intrinsic complexity of native soy protein systems, the aggregation mechanism differs depending on the extent of hydrolysis and on whether the substrate exists in its native or denatured state. Specifically, the differential aggregation behaviors among SPI, 7S (a highly hydrophobic trimer), and 11S (a disulfide-rich hexamer) during papain hydrolysis remain largely unknown. Comparing these three systems provides critical new mechanistic insights into how initial disulfide bond densities and varying degrees of exposed internal hydrophobic groups govern aggregate assembly.

Previous literature [1] demonstrated that in soy protein hydrolyzed by bromelain, aggregate formation was driven not only by hydrophobic interactions but also by disulfide bonds. During the proteolytic hydrolysis of soy protein, the newly generated peptide chains can interact via hydrophobic forces, which subsequently form the structural basis of complex gel networks [3]. Later, researchers at Kyoto University, using turbidity measurements, proposed that aggregation induced by *Bacillus* protease is primarily driven by hydrophobic interactions among newly exposed internal hydrophobic groups [4]. Kuipers et al. [5] further revealed that protein aggregation is not solely determined by the balance between electrostatic repulsion and hydrophobic attraction but is more complex, as specific electrostatic attractions may also be involved, rendering the mechanism more subtle and less predictable. Subsequent studies extended this line of research to whey protein hydrolysates. Otte et al. [6] suggested that electrostatic interactions contribute to the formation of β-lactoglobulin (β-LG) aggregates, whereas Doucet et al. [7] reported that the aggregation of whey protein peptides is primarily driven by hydrophobic interactions, with hydrogen bonding and electrostatic interactions playing only minor roles. Noiseux et al. [8] further reported that hydrophobic interactions are the predominant intermolecular forces driving the aggregation of whey β-LG hydrolysates produced by trypsin. Despite extensive efforts to elucidate the mechanisms of protein hydrolysate aggregation [9], the mechanism underlying soy protein aggregation remains inconclusive, with many aspects still unresolved [10,11]. Current evidence suggests that soy protein aggregation and gelation are primarily mediated by noncovalent interactions, particularly hydrophobic interactions, among hydrolyzed peptides [12].

Several studies have reported that peptides isolated from soy protein hydrolysates can inhibit platelet aggregation [13]. For instance, the addition of hydrolysates to dairy cheese was found to gradually reduce viscosity, while bioactive peptides derived from milk were shown to affect coagulation [14]. Barbeau et al. [15] further demonstrated that whey peptide fractions can thermally stabilize β-lactoglobulin. In particular, papain treatment of 11S soy protein produces gels formed by hydrophobic interactions among the peptides [16]. The ability of peptides to inhibit aggregation is of considerable importance for the food industry, especially in applications where protein aggregation is undesirable.

The present study addresses the problem of low supernatant protein yield during papain hydrolysis of soy protein. In food processing, controlling aggregation is often critical. To modulate this, chemical additives such as Na_2_SO_3_, cysteine, and SDS were evaluated. Na_2_SO_3_ and cysteine are strong reducing agents that can effectively cleave intra- and intermolecular disulfide bonds. SDS is a potent amphiphilic surfactant known to disrupt hydrophobic interactions. It should be noted that while SDS is generally unsuitable for direct food applications and serves strictly as a mechanistic probe in this study, cysteine represents a viable, food-compatible alternative with significant industrial relevance. Because these compounds were introduced immediately before enzyme inactivation, the study was specifically designed to test post-hydrolysis aggregation control rather than the prevention of aggregation throughout the entire enzymatic process. Using papain-hydrolyzed soy protein as a model system, 0.1% Na_2_SO_3_, 0.1% cysteine, and 0.1% SDS were added at the enzyme inactivation stage. Product yields were determined, and the intermolecular forces underlying aggregation were analyzed to assess the inhibitory effects of these additives on aggregate formation. This strategy aims to mitigate aggregation and precipitation mediated by hydrophobic interactions and disulfide bonds, thereby providing a theoretical basis for controlling protein aggregation during enzymatic hydrolysis.

## 2. Results and Discussion

### 2.1. Analysis of Intermolecular Forces Involved in the Aggregation Process

#### 2.1.1. Solubility Analysis of Aggregates

Under identical conditions, papain hydrolysis of different soy proteins (SPI, 7S, and 11S) led to the formation of aggregates. These aggregates exhibited low solubility in aqueous solution and were predominantly present as precipitates. The solubility of aggregates or precipitates in specific denaturing solvents reflects the intermolecular forces responsible for their formation [17,18]. Therefore, it was necessary to examine changes in turbidity of different aggregates after treatment with various concentrations of denaturants, in order to identify the types of interactions involved in the aggregation process. In these experiments, the protein concentration was maintained at 3 mg/mL.

To systematically evaluate the specific contributions of varying intermolecular forces, specific concentrations of denaturants (guanidine hydrochloride and urea) and reducing agents (β-Me) were chosen. The turbidity values of SPI, 11S and 7S hydrolysate aggregates treated with different concentrations of denaturants (guanidine hydrochloride, urea, and SDS) and reducing agents (β-Me) are presented in Table 1, Table 2 and Table 3. The turbidity of 11S aggregates (0.137) was significantly higher than that of SPI aggregates at the same protein concentration (0.114), indicating that 11S forms a greater number of aggregates during hydrolysis. The turbidity of precipitates from SPI, 7S, and 11S decreased with increasing concentrations of guanidine hydrochloride, urea, and SDS, whereas the reducing agent β-Me had little effect. Both 8 mol/L urea and 6 mol/L guanidine hydrochloride almost completely solubilized the aggregates, effectively disrupting hydrogen bond-stabilized protein conformations and weakening hydrophobic interactions. Notably, even a low concentration of SDS (0.1 mol/L) rapidly enhanced aggregate solubility. As an amphiphilic molecule, SDS contains both hydrophilic and hydrophobic groups, enabling it to exert electrostatic and hydrophobic interactions simultaneously, form a hydration layer on the molecular surface, and thereby solubilize aggregates effectively. In contrast, β-Me showed no significant effect even at 4 mol/L, suggesting that most intra- and intermolecular disulfide bonds in the hydrolysis aggregates had already been disrupted. The percentage decrease in turbidity of 11S aggregates in SDS and β-Me solutions was greater than that of SPI, suggesting that 11S aggregates contain more disulfide-linked fragments. Collectively, these results indicate that hydrophobic interactions play a dominant role in the enzymatic aggregation of SPI and 11S, whereas other noncovalent interactions may make auxiliary contributions, and covalent bonding is likely minimal. A reduction in OD660 primarily reflects the disruption of scattering particles rather than the absolute solubilization of all protein aggregates. It should also be noted that the 0.1% SDS (*w*/*v*) used in the subsequent additive experiment corresponds to a molar concentration of approximately 3.47 mmol/L. The use of molar concentrations (mol/L) in Table 1, Table 2 and Table 3 was chosen to perfectly align with standard high-concentration denaturant assays, whereas the 0.1% metric reflects practical weight/volume food processing parameters.

#### 2.1.2. Amino Acid Analysis

The amino acid composition of soy protein plays a critical role in determining its structural characteristics and functional properties, with hydrophobic amino acids exerting a particularly significant influence. The relative proportion of hydrophobic residues not only affects protein solubility and stability in aqueous solutions but also governs the strength of hydrophobic interactions within hydrolysates. During enzymatic hydrolysis, the exposure of hydrophobic residues can promote intermolecular interactions, thereby facilitating aggregate formation in the hydrolysate [19].

Table 4 presents the amino acid composition of the supernatants and aggregates obtained from the enzymatic hydrolysis of the three soy proteins. The numbers reported in the table represent the percentage content (g/100 g protein) of each amino acid relative to the total protein mass. Tryptophan was not detected because it is degraded during acid hydrolysis. The results show that the overall content of hydrophobic amino acids in the aggregates was relatively similar across the three proteins. Residues such as phenylalanine, isoleucine, leucine, valine, alanine, and methionine were enriched in the aggregates compared with the supernatants, whereas six hydrophilic amino acids (glutamic acid, aspartic acid, arginine, lysine, serine, and threonine) were present at lower levels. Histidine exhibited mixed trends depending on the specific protein. These findings suggest that hydrophobic interactions are highly correlated with hydrolysate aggregation, while hydrogen bonding and electrostatic interactions may also contribute to aggregate stabilization, which is consistent with the observations reported by Xu et al. [20]. However, it must be acknowledged that conventional acid hydrolysis carries inherent analytical limitations; because specific oxidation/protection procedures were not employed, sulfur-containing amino acids such as methionine and cysteine may be partially altered or degraded. Furthermore, while the enrichment of hydrophobic amino acids strongly correlates with hydrophobic interactions, alternative mechanisms—such as differences in peptide size distribution and differential peptide solubility among the fractions—cannot be entirely ruled out.

#### 2.1.3. Determination of Sulfhydryl Content

To date, the enzymatic hydrolysis-induced aggregation of soy protein has been extensively investigated; however, the role of sulfhydryl group dynamics during hydrolysis in driving aggregation remains insufficiently understood. Protein hydrolysates constitute a complex system in which interactions between sulfhydryl groups and disulfide bonds can promote the aggregation of specific peptide fragments, leading to the formation of high-molecular-weight precipitates. This process subsequently alters the distribution of sulfhydryl and disulfide contents between the supernatant and precipitate, thereby providing a theoretical framework for understanding the properties of hydrolysates, optimizing hydrolysis conditions, and guiding their practical applications. Before hydrolysis, the total sulfhydryl contents of unhydrolyzed SPI, 7S, and 11S proteins were determined to be 7.57, 6.96, and 7.28 μmol/g protein, respectively.

As shown in Table 5, the free and total sulfhydryl contents were highest in SPI hydrolysates, followed by 11S, while 7S exhibited the lowest levels. Different lowercase letters (a and b) reported in the table indicate significant statistical differences (*p* < 0.05) among the different samples. In 7S hydrolysates, both the supernatant and aggregate fractions showed a decrease in total sulfhydryl content compared with the unhydrolyzed protein, whereas in 11S hydrolysates, the total sulfhydryl content increased. This difference may be explained by the stronger susceptibility of 7S to papain, where extensive cleavage at a hydrolysis degree of 5% promotes protein aggregation. During this process, some sulfhydryl groups may undergo oxidation (forming disulfide bonds or other derivatives) or desulfhydration, leading to a reduction in total sulfhydryl content [20]. By contrast, mild hydrolysis of 11S induces partial unfolding of the protein structure, disrupts disulfide bonds, and exposes sulfhydryl groups on the molecular surface, thereby increasing both total and free sulfhydryl contents. For SPI, 7S, and 11S, the sulfhydryl content in the supernatant was consistently lower than that in the precipitate, suggesting that disulfide bonds play a role in aggregate formation. Although the reducing agent β-Me showed little effect on pre-formed aggregates in the earlier turbidity assay (Section 2.1.1)—likely because the tightly packed hydrophobic networks physically hindered β-Me from accessing buried disulfide bonds—the direct sulfhydryl measurements effectively capture the dynamic intra- and intermolecular disulfide shuffling occurring during the active hydrolysis process, thereby reconciling these observations [21]. Furthermore, while total and free sulfhydryl measurements cannot directly quantify absolute disulfide concentrations, the relative differences between supernatant and precipitate provide a robust indirect index of dynamic disulfide involvement. To ensure analytical accuracy, strict sample-specific blanks were utilized for measurements at 412 nm to completely eliminate any optical interference from denaturants such as guanidine hydrochloride.

### 2.2. Effects of Additives on the Properties of Hydrolysates

#### 2.2.1. Yield of Hydrolysate Supernatant

Both Na_2_SO_3_ and cysteine (Cys) are strong reducing agents, with Cys being more potent, and therefore have a high capacity to cleave disulfide bonds. In contrast, SDS functions as a surfactant that can disrupt hydrophobic interactions under suitable conditions. Aggregation in the hydrolysate was observed to be influenced by the enzyme inactivation stage. Accordingly, 0.1% of each blocking agent was added prior to enzyme inactivation to evaluate their effectiveness in controlling enzymatic aggregation.

**Figure 1:**
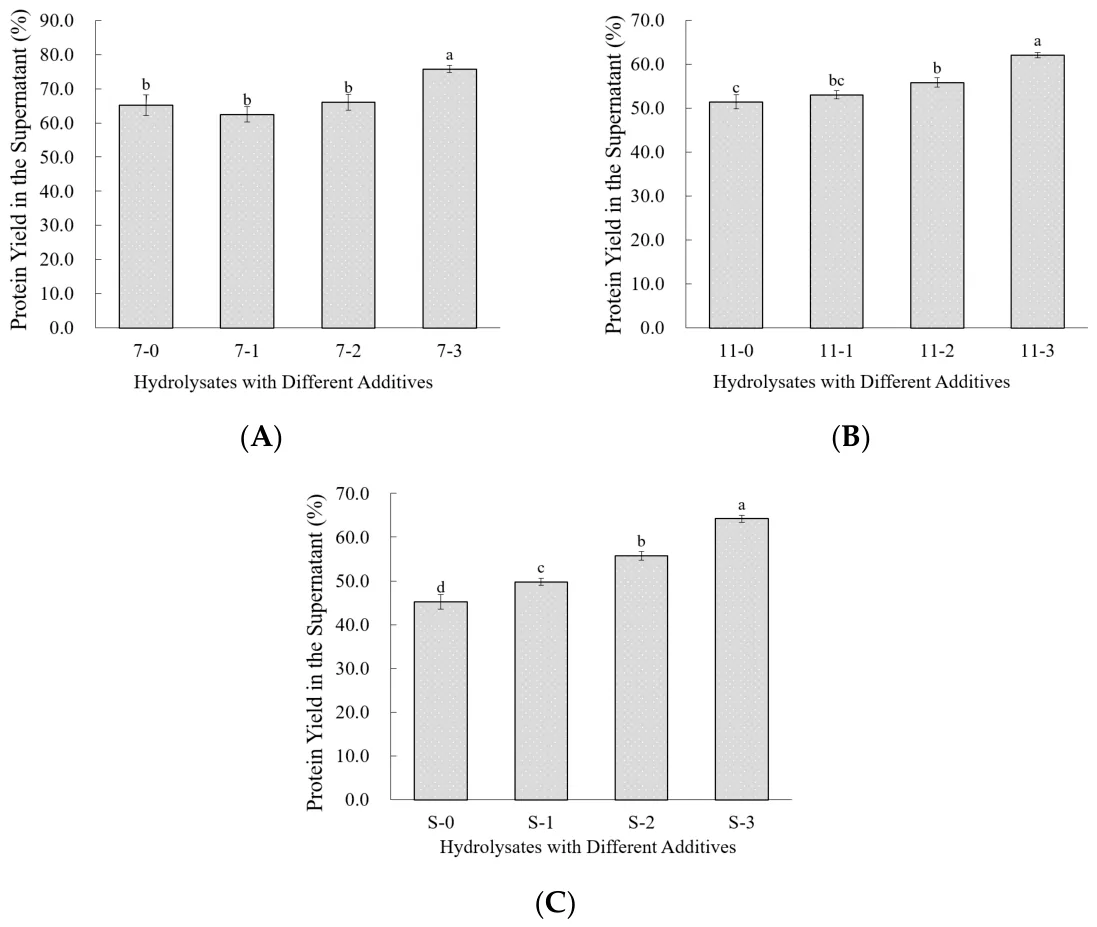
Effects of additives on the supernatant yield from soy protein hydrolysates: (**A**) 7S, (**B**) 11S, and (**C**) soy protein isolate (SPI). Data are presented as mean ± standard deviation (SD) of three replicates (*n* = 3). Different lowercase letters indicate significant differences among treatments within each protein fraction (*p* < 0.05).

As shown in Figure 1, The hydrolysates were then centrifuged, and the supernatants were collected to determine the supernatant yield. Overall, samples treated with 0.1% cysteine (Cys) exhibited higher supernatant yields than those treated with 0.1% Na_2_SO_3_. For all proteins (7S, 11S, and SPI), the addition of SDS produced the greatest increase in supernatant yield compared with the control, with absolute percentage-point increases of 12.57%, 10.73%, and 18.77%, respectively. For 7S, the increase in supernatant yield upon addition of 0.1% SDS was greater than that of 11S, whereas for 11S, addition of 0.1% Cys resulted in a greater increase in supernatant yield compared with 7S. This may be attributed to the higher content of hydrophobic amino acid residues in 7S and the greater number of disulfide bonds in 11S [16]. Collectively, these findings indicate that hydrophobic interactions are the predominant driving force in the aggregation of SPI and 11S hydrolysates, with additional contributions from disulfide-linked fragments, whereas aggregation of 7S hydrolysates is mainly governed by hydrophobic interactions.

#### 2.2.2. Tricine-SDS-PAGE Analysis

**Figure 2:**
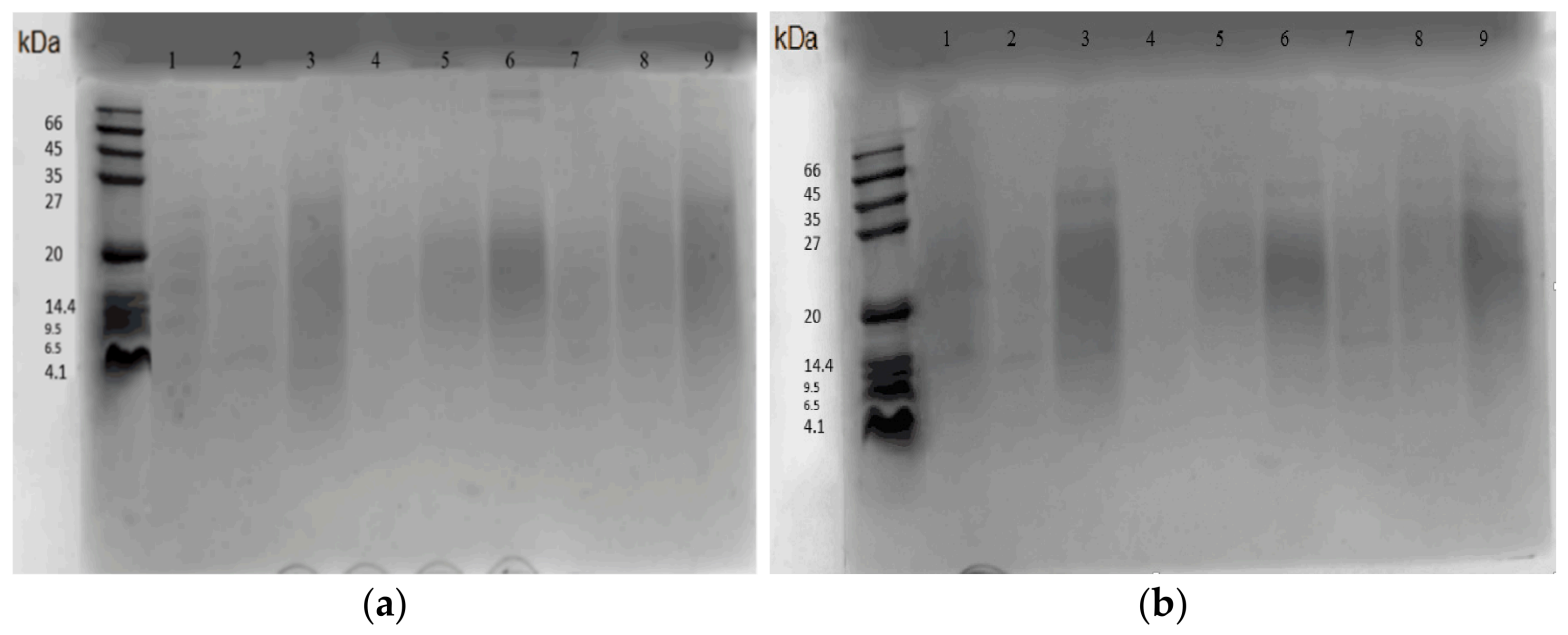
Reducing and non-reducing Tricine-SDS-PAGE patterns of supernatant fluid: (**a**) non-reducing; (**b**) reducing. Lanes 1–3, 4–6, and 7–9 correspond to the hydrolysate supernatants of 7S, SPI, and 11S, respectively, treated with 0.1% Na_2_SO_3_ (Lanes 1, 4, 7), 0.1% cysteine (Lanes 2, 5, 8), and 0.1% SDS (Lanes 3, 6, 9).

**Figure 3:**
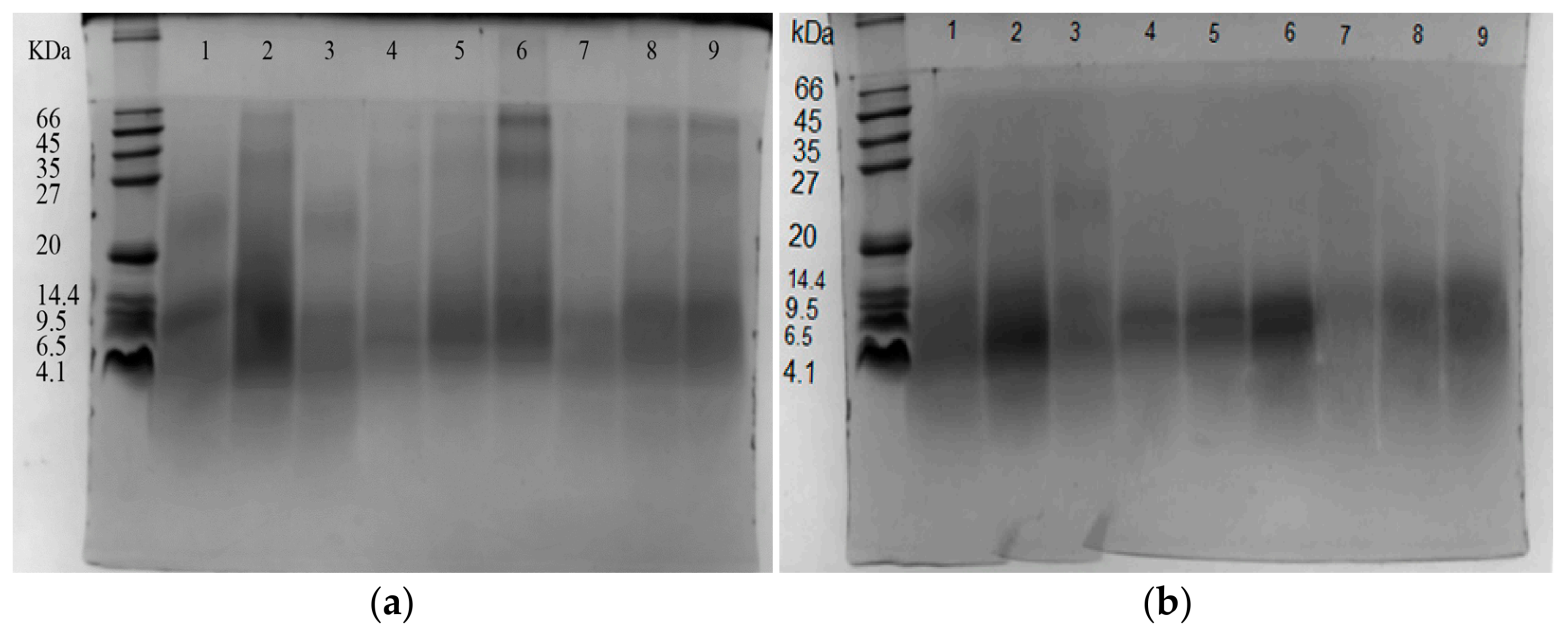
Reducing and non-reducing Tricine-SDS-PAGE patterns of precipitates: (**a**) non-reducing; (**b**) reducing. Lanes 1–3, 4–6, and 7–9 correspond to the hydrolysate precipitates of 7S, SPI, and 11S, respectively, treated with 0.1% Na_2_SO_3_ (Lanes 1, 4, 7), 0.1% cysteine (Lanes 2, 5, 8), and 0.1% SDS (Lanes 3, 6, 9).

To further examine the effects of different additives on the enzymatic aggregation of soy protein, hydrolysates with a degree of hydrolysis (DH) of 5% were prepared using papain. The resulting supernatants and precipitates were analyzed by Tricine-SDS-PAGE under both non-reducing and reducing conditions. The results are presented in Figure 2 and Figure 3.

Analysis of the non-reducing and reducing Tricine-SDS-PAGE profiles of hydrolysate supernatants and precipitates revealed that, irrespective of the blocking agent used, the supernatant compositions were largely similar, whereas the precipitates exhibited marked compositional differences. This suggests that disulfide-linked fragments were essentially absent in the supernatants but present to varying degrees in the precipitates. Comparison of Figure 2a and Figure 3a showed that the molecular weight distribution in the precipitates was nearly continuous, with a substantially higher proportion of fragments below 20 kDa compared with the supernatants, confirming that the precipitates were primarily formed through the aggregation of low-molecular-weight peptides. Lanes 1, 2, and 3 exhibited no apparent differences before and after reduction, while lanes 5, 6, 8, and 9 displayed more pronounced compositional changes. Based on band migration and staining intensity, it can be inferred that hydrolysates treated with 0.1% cysteine (Cys) contained virtually no disulfide-linked fragments after centrifugation, whereas precipitates from hydrolysates treated with 0.1% SDS showed substantial differences between non-reducing and reducing conditions. For 7S, no significant differences were observed between the two conditions, whereas 11S exhibited pronounced changes upon reduction. It should be noted that highly cross-linked, high-molecular-weight materials may not enter the resolving gel; thus, the absence of visible high-molecular-weight bands does not necessarily indicate the complete absence of large aggregates. Furthermore, densitometric quantification of major bands was not performed, which represents an inherent limitation of this visual comparative analysis.

## 3. Materials and Methods

### 3.1. Materials and Reagents

Northeast soybeans were purchased from Qingmei Seed Industry Co., Ltd. (Heilongjiang, China). Papain (enzyme activity: 800 U/mg) was obtained from Shanghai Ruji Biotechnology Co., Ltd. (Shanghai, China). Folin–Ciocalteu reagent (2N, analytical grade), 30% acrylamide solution (19:1), 30% acrylamide solution (29:1), tetramethylethylenediamine (TEMED), dithiothreitol (DTT), Coomassie Brilliant Blue R-250, Coomassie Brilliant Blue G-250, 5,5′-dithiobis-(2-nitrobenzoic acid) (DTNB), and 1-anilinonaphthalene-8-sulfonate (ANS) were purchased from Sigma (St. Louis, MO, USA). All other reagents were of analytical grade and obtained from Sinopharm Chemical Reagent Co., Ltd. (Shanghai, China).

### 3.2. Instruments and Equipment

An RW20D stirrer (IKA Laboratory Technology, Staufen, Germany); DC10 thermostatic water bath (Thermo, Dreieich, Germany); AB104-N digital electronic balance (Mettler Toledo Instruments Co., Ltd., Shanghai, China); DHG-9070 thermostatic drying oven (Yiheng Scientific Instruments Co., Ltd., Shanghai, China); 3K15 benchtop refrigerated centrifuge (Sigma, USA); UV2800 UV–Vis spectrophotometer (Unico Instruments Co., Ltd., Shanghai, China); Mini-PROTEAN III gel electrophoresis system (Bio-Rad, Hercules, CA, USA); Avanti J-26 high-speed centrifuge (Beckman Coulter, Brea, CA, USA); and Agilent 1100 high-performance liquid chromatography system (Agilent Technologies, Santa Clara, CA, USA) were used.

### 3.3. Isolation of 7S, 11S, and SPI

SPI, 7S, and 11S globulins were extracted from soybeans using standard isoelectric precipitation and fractionation protocols based on their specific solubilities at distinct pH levels. Briefly, defatted soy flour was dispersed in deionized water (1:10, *w*/*v*) and the pH was adjusted to 8.0 using 2 mol/L NaOH, followed by 2 h of continuous stirring at room temperature. After centrifugation (10,000× g, 20 min), the supernatant was adjusted to pH 4.5 using 2 mol/L HCl to precipitate the whole SPI. For fractionation, the initial pH 8.0 aqueous extract was instead adjusted to pH 6.4 to preferentially precipitate 11S globulin overnight at 4 °C. The remaining supernatant was subsequently recovered and adjusted to pH 4.8 to precipitate the 7S globulin. The purity of the isolated 7S and 11S fractions was confirmed to be >85% using preliminary SDS-PAGE analysis.

### 3.4. Preparation of Hydrolysates with Different Additives

Solutions of 7S, 11S, and SPI were prepared at a concentration of 5% (*w*/*v*) in 0.05 M phosphate buffer (pH 7.0). For each assay, 100 mL of solution was transferred into a reaction vessel, the pH was adjusted to 7.0, and the temperature was maintained at 55 °C. Prior to hydrolysis, the solutions were stirred for 30 min. Papain (800 U/mg) was preactivated in the reaction buffer at 37 °C for 15 min, after which it was added at an enzyme-to-substrate ratio of 1:100 (*w*/*w*). The hydrolysis reaction was carried out for 120 min. Hydrolysis proceeded rapidly, during which 0.2 mol/L NaOH was continuously titrated to maintain a constant pH, and NaOH consumption was recorded every minute. The degree of hydrolysis (DH) was actively and continuously monitored using the standard pH-stat method. Once a uniform DH of exactly 5% was achieved for all three substrates under these standardized conditions, the targeted additives were introduced. To examine the effects of different additives on hydrolysis-induced aggregation, 0.1% Na_2_SO_3_, 0.1% cysteine (Cys), or 0.1% sodium dodecyl sulfate (SDS) (*w*/*v*) was added immediately prior to enzyme inactivation. The enzyme was then inactivated by heating the mixture in a boiling water bath for 10 min. Samples were designated as follows: 7-0, 11-0, and S-0 for hydrolysates without additives; 7-1, 11-1, and S-1 for hydrolysates with 0.1% Na_2_SO_3_; 7-2, 11-2, and S-2 for hydrolysates with 0.1% Cys; and 7-3, 11-3, and S-3 for hydrolysates with 0.1% SDS. Following inactivation, samples were centrifuged (10,000× *g*, 15 min), and both the resulting supernatant and precipitate were carefully separated, collected, and stored at 4 °C prior to immediate subsequent analysis.

### 3.5. Tricine-SDS-PAGE

Tricine-SDS-PAGE was performed according to the method of Schägger [22] with minor modifications.

#### 3.5.1. Reagent Preparation

The 2× loading buffer was prepared by mixing 1 mol/L Tris-HCl (pH 6.87, 2 mL), glycerol (3 g), SDS (0.8 g), DTT (0.31 g), and Coomassie Brilliant Blue G-250 (0.002 g). The components were dissolved in ultrapure water, adjusted to a final volume of 10 mL, and stored at −20 °C.

#### 3.5.2. Procedure

Polyacrylamide gels were prepared, and protein samples were adjusted to a concentration of 2 mg/mL. The samples were heated in a boiling water bath for 5 min, and 8 μL of each sample was loaded. Electrophoresis was performed under the following conditions: stacking gel, 50 V; spacer gel, 80 V; and separating gel, 120 V.

### 3.6. Amino Acid Analysis

Amino acid analysis was performed according to the method of Otter [23] with slight modifications. Approximately 100 mg of sample was accurately weighed into a custom hydrolysis tube and dissolved in 8 mL of 6 mol/L HCl. After flushing with nitrogen for 3 min to remove air, the tube was immediately sealed and hydrolyzed in an oven at 120 °C for 22 h. Following hydrolysis, 4.8 mL of 10 mol/L NaOH was added, and the solution was transferred to a 25 mL volumetric flask and diluted to volume. The mixture was filtered through double-layer filter paper, and the filtrate was collected in centrifuge tubes for further use. For analysis, 1 mL of filtrate was centrifuged for 10 min in a 1.5 mL tube, and 400 μL of the resulting supernatant was transferred to sample vials and labeled. Pre-column derivatization was performed with *o*-phthalaldehyde (OPA), and *N-*(9-fluorenylmethoxycarbonyl) chloride (FMOC-Cl) was used for secondary amino acids (proline and hydroxyproline). Amino acid contents were quantified using the external standard method via High-Performance Liquid Chromatography (HPLC) coupled with a fluorescence detector using a standard mixture of 17 amino acids (Sigma-Aldrich, St. Louis, MO, USA).

### 3.7. Determination of Sulfhydryl Content

Sulfhydryl groups were determined using Ellman’s reagent method [24]. Briefly, 1 mL of protein solution was mixed with 4 mL of 0.1 mol/L phosphate buffer (pH 8.0), followed by the addition of 0.05 mL of 0.01 mol/L 5,5′-dithiobis-(2-nitrobenzoic acid) (DTNB) (prepared fresh in 0.1 mol/L phosphate buffer, pH 8.0). The mixture was vortexed and incubated at room temperature (25 °C) for 20 min, after which the absorbance was measured at 412 nm. For determination of total sulfhydryl content, the phosphate buffer was replaced with 0.1 mol/L phosphate buffer (pH 8.0) containing 6 mol/L guanidine hydrochloride, while all other procedures remained unchanged. Sulfhydryl content (µmol/g protein) was calculated using a molar extinction coefficient of 13,600 M^−1^·cm^−1^. To completely eliminate potential optical interference from denaturants like guanidine hydrochloride, rigorous sample-specific blanks were applied for all absorbance measurements at 412 nm.

### 3.8. Determination of Turbidity

The turbidity of the aggregates in the presence of different denaturants (including varying concentrations of urea from 0.5 to 8 mol/L, guanidine hydrochloride from 0.5 to 6 mol/L, SDS from 0.1 to 2 mol/L, and β-Me from 0.5 to 4 mol/L) was determined by measuring the optical density at 660 nm (OD_660nm_) using a UV–Vis spectrophotometer as described in previous literature [21]. The protein concentration was maintained at 3 mg/mL during all measurements.

### 3.9. Statistical Analysis

All experiments were performed with at least three independent biological replicates (*n* = 3), and data are expressed as the mean ± standard deviation (SD). Statistical analysis was performed using SPSS version 25.0 (SPSS Inc., Chicago, IL, USA). Prior to significance testing, the assumptions of normality and homogeneity of variance for the datasets were rigorously verified using the Shapiro–Wilk test and Levene’s test, respectively. Data were analyzed by one-way analysis of variance (ANOVA) followed by Duncan’s post-hoc multiple range test to determine significant differences between treatment means. Differences were considered statistically significant at *p* < 0.05.

## 4. Conclusions

The intermolecular forces driving the enzymatic aggregation of soy proteins were first investigated through assessments of aggregate solubility, amino acid composition, and sulfhydryl content. Subsequently, strategies to control hydrolysate aggregation during the enzyme inactivation stage were evaluated using supernatant yield measurements and electrophoretic analyses. The results demonstrated that the turbidity of SPI, 7S, and 11S aggregates decreased with increasing concentrations of denaturants (guanidine hydrochloride, urea, and SDS), whereas the reducing agent β-mercaptoethanol (β-Me) had little effect. The higher hydrophobic amino acid content in the precipitates compared with the supernatants indicated that hydrophobic interactions are a major contributing factor in enzymatic aggregation. Analysis of sulfhydryl contents revealed a decrease in both supernatants and precipitates of 7S hydrolysates relative to the unhydrolyzed protein, whereas an increase was observed in 11S hydrolysates. For all three proteins, sulfhydryl levels in the supernatants were consistently lower than in the precipitates, suggesting an auxiliary contribution of disulfide bonds to aggregate formation. The addition of 0.1% SDS to 7S, 11S, and SPI hydrolysates produced the greatest improvement in supernatant yield compared with the control, with increases of 12.57%, 10.73%, and 18.77%, respectively. Collectively, these findings indicate that aggregation of SPI and 11S hydrolysates involve strong hydrophobic interactions together with auxiliary contributions from disulfide-linked fragments, whereas aggregation of 7S hydrolysates is driven predominantly by hydrophobic interactions.

## Figures and Tables

**Table 1:** Turbidity of aggregates of SPI treated with different concentrations of dissociating reagents (*n* = 3).

Molar Concentration (mol/L)		Turbidity (OD_660nm_)		
	Urea	Guanidine Hydrochloride	SDS	β-Me
0	0.114 ± 0.005 ^a^	0.114 ± 0.005 ^a^	0.114 ± 0.005 ^a^	0.114 ± 0.005 ^a^
0.5	0.108 ± 0.009 ^ab^	0.109 ± 0.005 ^a^	0.031 ± 0.001 ^b^	0.114 ± 0.007 ^a^
1	0.084 ± 0.007 ^bc^	0.089 ± 0.008 ^b^	0.015 ± 0.001 ^c^	0.113 ± 0.001 ^a^
2	0.053 ± 0.004 ^c^	0.063 ± 0.005 ^c^	0.015 ± 0.001 ^c^	0.110 ± 0.001 ^ab^
4	0.040 ± 0.001 ^d^	0.033 ± 0.001 ^d^		0.106 ± 0.005 ^b^
6	0.021 ± 0.001 ^e^	0.019 ± 0.001 ^e^		
8	0.019 ± 0.001 ^e^			

Note: Data are presented as mean ± SD (*n* = 3). Different lowercase letters (a–e) within the same column indicate statistically significant differences (*p* < 0.05) among different molar concentrations based on Duncan’s multiple range test.

**Table 2:** Turbidity of aggregates of 11S treated with different concentrations of dissociating reagents (*n* = 3).

Molar Concentration (mol/L)		Turbidity (OD_660nm_)		
	Urea	Guanidine Hydrochloride	SDS	β-Me
0	0.137 ± 0.005 ^a^	0.137 ± 0.005 ^a^	0.137 ± 0.005 ^a^	0.137 ± 0.005 ^a^
0.5	0.128 ± 0.006 ^ab^	0.125 ± 0.005 ^b^	0.068 ± 0.003 ^b^	0.136 ± 0.004 ^a^
1	0.104 ± 0.003 ^c^	0.109 ± 0.003 ^c^	0.019 ± 0.001 ^c^	0.130 ± 0.001 ^ab^
2	0.078 ± 0.003 ^d^	0.088 ± 0.003 ^d^	0.019 ± 0.001 ^c^	0.124 ± 0.002 ^bc^
4	0.039 ± 0.001 ^e^	0.034 ± 0.001 ^e^	0.009 ± 0.001 ^d^	0.121 ± 0.001 ^c^
6	0.024 ± 0.001 ^f^	0.027 ± 0.001 ^f^		
8	0.023 ± 0.001 ^f^			

Note: Data are presented as mean ± SD (*n* = 3). Different lowercase letters (a–f) within the same column indicate statistically significant differences (*p* < 0.05) among different molar concentrations based on Duncan’s multiple range test.

**Table 3:** Turbidity of aggregates of 7S treated with different concentrations of dissociating reagents (*n* = 3).

Molar Concentration (mol/L)		Turbidity (OD_660nm_)		
	Urea	Guanidine Hydrochloride	SDS	β-Me
0	0.107 ± 0.005 ^a^	0.107 ± 0.008 ^a^	0.107 ± 0.005 ^a^	0.107 ± 0.006 ^a^
0.5	0.102 ± 0.010 ^ab^	0.094 ± 0.012 ^ab^	0.058 ± 0.003 ^b^	0.104 ± 0.004 ^a^
1	0.086 ± 0.001 ^bc^	0.075 ± 0.006 ^bc^	0.018 ± 0.002 ^c^	0.099 ± 0.002 ^ab^
2	0.068 ± 0.003 ^c^	0.054 ± 0.003 ^c^	0.018 ± 0.001 ^c^	0.092 ± 0.002 ^b^
4	0.025 ± 0.004 ^d^	0.021 ± 0.002 ^d^		0.094 ± 0.001 ^b^
6	0.021 ± 0.002 ^de^	0.017 ± 0.001 ^d^		
8	0.019 ± 0.001 ^e^			

Note: Data are presented as mean ± SD (*n* = 3). Different lowercase letters (a–e) within the same column indicate statistically significant differences (*p* < 0.05) among different molar concentrations based on Duncan’s multiple range test.

**Table 4:** Amino acid composition (g/100 g protein) in the supernatant fluid and precipitates of enzymatic hydrolysates.

Amino Acid	7S		11S		SPI	
	Supernatant	Precipitate	Supernatant	Precipitate	Supernatant	Precipitate
Asp	7.96	9.10	8.88	7.92	7.22	6.68
Glu	19.40	13.10	19.00	12.50	14.90	9.29
Ser	2.99	3.00	3.11	2.78	2.57	2.41
His	1.66	2.07	1.75	1.54	1.49	1.68
Gly	2.40	2.39	3.23	3.00	2.62	2.46
Thr	1.32	1.55	1.84	1.83	1.60	1.64
Arg	6.52	6.01	5.82	5.52	5.09	4.44
Ala	2.14	2.72	2.40	2.89	2.27	2.66
Tyr	1.75	2.31	1.77	2.52	1.78	2.21
Cys	0.44	0.50	0.81	0.85	0.85	0.76
Val	3.03	3.74	3.26	4.03	2.87	3.51
Met	1.53	2.39	2.28	2.76	2.29	2.64
Phe	3.86	4.75	3.27	3.87	2.82	3.56
Ile	3.05	4.30	3.07	3.60	2.70	3.44
Leu	4.75	7.23	4.84	5.94	4.10	5.84
Lys	5.01	4.34	4.29	2.85	4.23	3.31
Pro	4.04	3.09	4.97	3.16	4.03	2.79

**Table 5:** Free and total sulfhydryl contents of the supernatant and precipitate fractions (*n* = 3).

Sample	Free Sulfhydryl Content (µmol/g Protein)			Total Sulfhydryl Content (µmol/g Protein)		
	SPI	7S	11S	SPI	7S	11S
Supernatant	7.17 ± 0.16 ^a^	4.19 ± 0.21 ^b^	4.34 ± 0.31 ^b^	8.60 ± 0.10 ^a^	6.10 ± 0.36 ^b^	8.21 ± 0.88 ^b^
Precipitate	7.57 ± 0.21 ^a^	5.59 ± 0.10 ^b^	4.89 ± 0.57 ^b^	8.75 ± 1.04 ^a^	6.76 ± 1.66 ^b^	8.08 ± 0.52 ^b^

Note: Data are presented as mean ± SD (*n* = 3). Different lowercase letters (a, b) in the same row within the “Free” or “Total” category indicate statistically significant differences (*p* < 0.05) among the different soy protein fractions (SPI, 7S, and 11S).

## Data Availability

Data are contained within the article.

## References

[1] Mohri M., Matsushita S. (1984). Improvement of Water Absorption of Soybean Protein by Treatment with Bromelain. J. Agric. Food Chem..

[2] Renkema J.M.S., Knabben J.H.M., Van Vliet T. (2001). Gel Formation by β-Conglycinin and Glycinin and Their Mixtures. Food Hydrocoll..

[3] Sekiguchi M., Matsuoka H., Fuke Y. (1985). Nature of Stem Bromelain Treatments on the Aggregation and Gelation of Soybean Protein. J. Food Sci..

[4] Nagai K., Inouye K. (2004). Insights into the Reaction Mechanism of the Coagulation of Soy Protein Isolates Induced by Subtilisin Carlsberg. J. Agric. Food Chem..

[5] Kuipers B.J.H., Van Koningsveld G.A., Alting A.C., Driehuis F., Gruppen H., Voragen A.G.J. (2005). Enzymatic Hydrolysis as a Means of Expanding the Cold Gelation Conditions of Soy Proteins. J. Agric. Food Chem..

[6] Otte J., Lomholt S.B., Halkier T., Qvist K.B. (2000). Identification of Peptides in Aggregates Formed during Hydrolysis of β-Lactoglobulin B with a Glu and Asp Specific Microbial Protease. J. Agric. Food Chem..

[7] Doucet D., Otter D.E., Gauthier S.F., Foegeding E.A. (2003). Enzyme-Induced Gelation of Extensively Hydrolyzed Whey Proteins by Alcalase: Peptide Identification and Determination of Enzyme Specificity. J. Agric. Food Chem..

[8] Noiseux I., Gauthier S.F., Turgeon S.L. (2002). Interactions between Bovine β-Lactoglobulin and Peptides under Different Physicochemical Conditions. J. Agric. Food Chem..

[9] Ipsen R., Otte J., Lomholt S.B., Qvist K.B. (2000). Standardized Reaction Times Used to Describe the Mechanism of Enzyme-Induced Gelation in Whey Protein Systems. J. Dairy Res..

[10] Chen W., Liang G., Li X., He Z., Zeng M., Gao D., Qin F., Goff H.D., Chen J. (2019). Effects of Soy Proteins and Hydrolysates on Fat Globule Coalescence and Meltdown Properties of Ice Cream. Food Hydrocoll..

[11] Kuipers B.J.H., Gruppen H. (2008). Identification of Strong Aggregating Regions in Soy Glycinin upon Enzymatic Hydrolysis. J. Agric. Food Chem..

[12] Li Z., Xie A., Yan Y., Piao C., Lan A., Wang S., Wu J., Fang X. (2025). Impacts of Soy Protein Isolate Hydrolysates on Gel Properties of High-Protein Soy Yogurt. Food Chem..

[13] Lee K.-A., Kim S.-H. (2005). SSGE and DEE, New Peptides Isolated from a Soy Protein Hydrolysate That Inhibit Platelet Aggregation. Food Chem..

[14] Rutherfurd K.J., Gill H.S. (2000). Peptides Affecting Coagulation. Br. J. Nutr..

[15] Barbeau J., Gauthier S.F., Pouliot Y. (1996). Thermal Stabilization of β-Lactoglobulin by Whey Peptide Fractions. J. Agric. Food Chem..

[16] Zhong F., Yang X., Li Y., Shoemaker C.F. (2006). Papain-induced Gelation of Soy Glycinin (11S). J. Food Sci..

[17] Yang Z., Liu N., Liu Y., Zhang B., Zhao M., Wang X., Wang X. (2023). Formation, Structure and Functional Characteristics of Amyloid Fibrils Formed Based on Soy Protein Isolates. Int. J. Biol. Macromol..

[18] Zhai X., Huang Y., Kong X., Li X., Chen Y., Zhang C. (2022). Characterization of Proteases from Irpex Lacteus Grown on Minimally Denatured Soybean Meals. J. Sci. Food Agric..

[19] Li Z., Ren J.M., Zhu L., Mohamed M.S., Wang Z. (2023). Gel Properties and Structural Characteristics of Composite Gels of Soy Protein Isolate and Silver Carp Protein. Gels.

[20] Xu X., Zhao H., Yuan B., Yan S., Li Y. (2025). Effect of Different Enzyme Pretreatment on the Formation and Gel Properties of Soy Protein Nanofibrils. Food Biosci..

[21] Pan N., Wan W., Du X., Kong B., Liu Q., Lv H., Xia X., Li F. (2021). Mechanisms of Change in Emulsifying Capacity Induced by Protein Denaturation and Aggregation in Quick-Frozen Pork Patties with Different Fat Levels and Freeze–Thaw Cycles. Foods.

[22] Schägger H. (2006). Tricine–SDS-PAGE. Nat. Protoc..

[23] Otter D.E. (2012). Standardised Methods for Amino Acid Analysis of Food. Br. J. Nutr..

[24] Hoshi Y., Yamauchi F. (2014). Determination of Sulfhydryl and Disulfide Contents of Soybean 11S Globulin and Their Change by Lyophilization. Agric. Biol. Chem..

